# Association of Autoimmune Hashimoto's Thyroiditis with Systemic Lupus Erythematosis

**DOI:** 10.7759/cureus.7261

**Published:** 2020-03-13

**Authors:** Chetan B Kammari, Subba Rao Daggubati, Venu Madhav Konala, Sreedhar Adapa, Srikanth Naramala

**Affiliations:** 1 Hospitalist, Cape Fear Valley Hospital, Fayetteville, USA; 2 Family Medicine / Hospitalist, Wise Health System, Decatur, USA; 3 Hematology and Oncology, Ashland Bellefonte Cancer Center, Ashland, USA; 4 Hematology and Oncology, Kings Daughters Medical Center, Ashland, USA; 5 Nephrology, Kaweah Delta Medical Center, Visalia, USA; 6 Rheumatology, Adventist Medical Center, Hanford, USA

**Keywords:** sle, hashimotos thyroiditis, pleural effusion, pericardial effusion

## Abstract

SLE (systemic lupus erythematosus) can be associated with other autoimmune disorders with overlapping clinical symptoms. We present a case of a 22-year-old male with recurring exertional dyspnea, chest pain, dry cough and chills, which on further testing revealed large pericardial effusion and bilateral pleural effusions along with laboratory abnormalities consistent with a diagnosis of overlap of SLE with serositis and Hashimoto's thyroiditis. SLE patients with underlying hypothyroidism are slow to respond to standard therapy unless the underlying hypothyroidism is adequately treated.

## Introduction

Pericardial and pleural effusions are some of the manifestations of serositis in SLE (systemic lupus erythematosus). Patients with SLE also have other associated autoimmune disorders with overlapping clinical symptoms. Our case highlights the importance of evaluating other differentials, especially hypothyroidism, which could also be present concurrently and contribute to the symptoms. SLE patients with underlying hypothyroidism are slow to respond to standard therapy unless the underlying hypothyroidism is adequately treated [1].

## Case presentation

**Figure 1:**
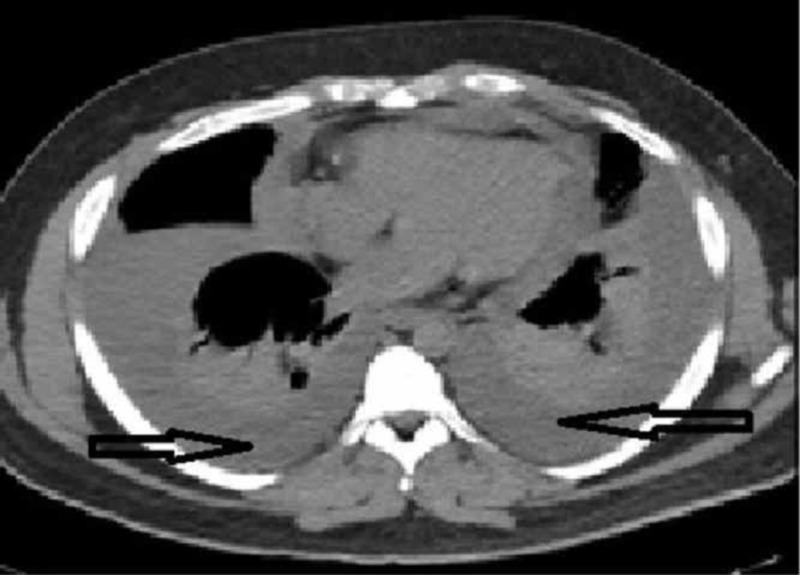
Computed tomography of chest (mediastinal window) showing pericardial effusion and bilateral pleural effusion Arrows pointing pleural and pericardial effusion

**Figure 2:**
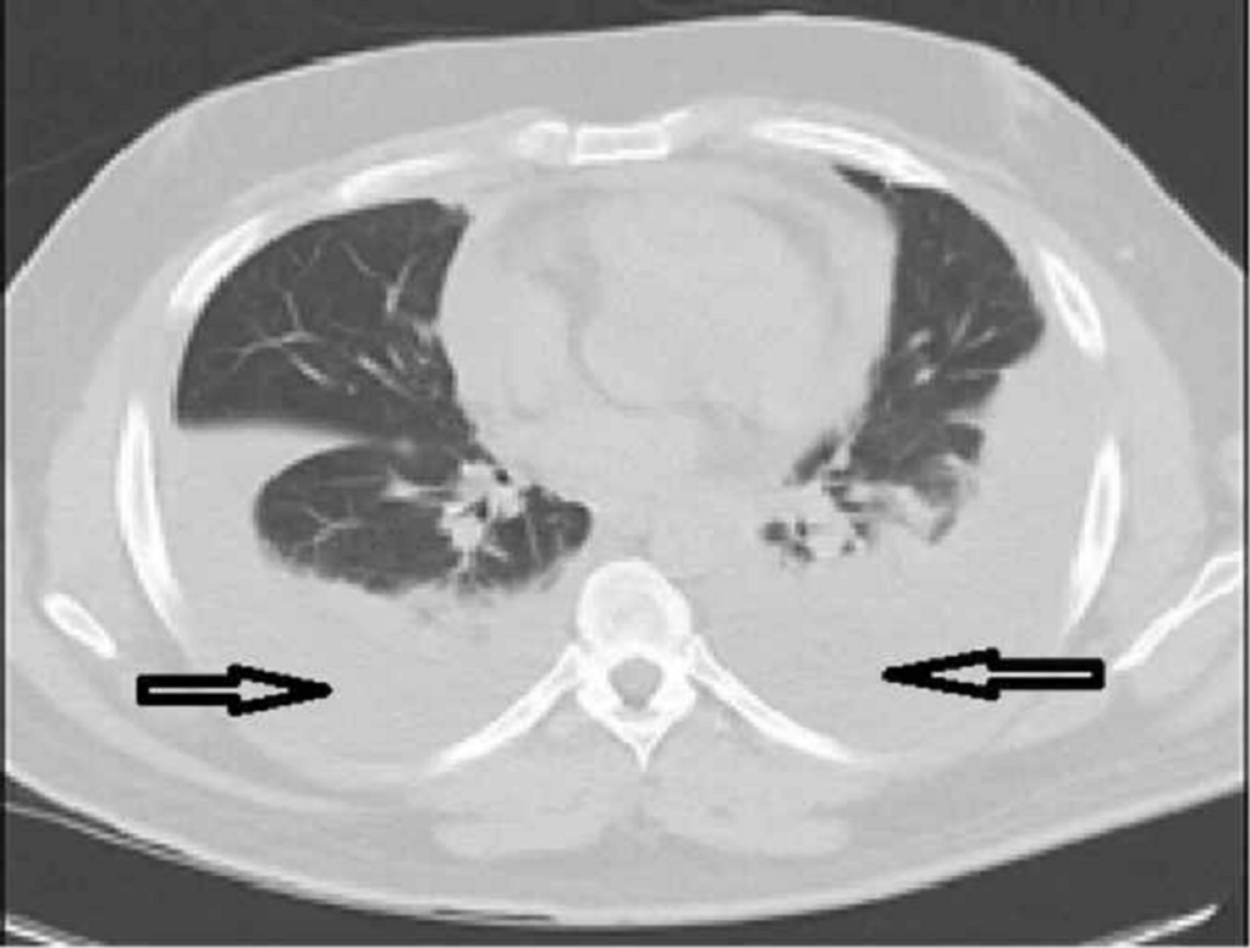
Computed tomography of chest (lung window) showing pericardial effusion and bilateral pleural effusion Arrows pointing pleural and pericardial effusion

**Figure 3:**
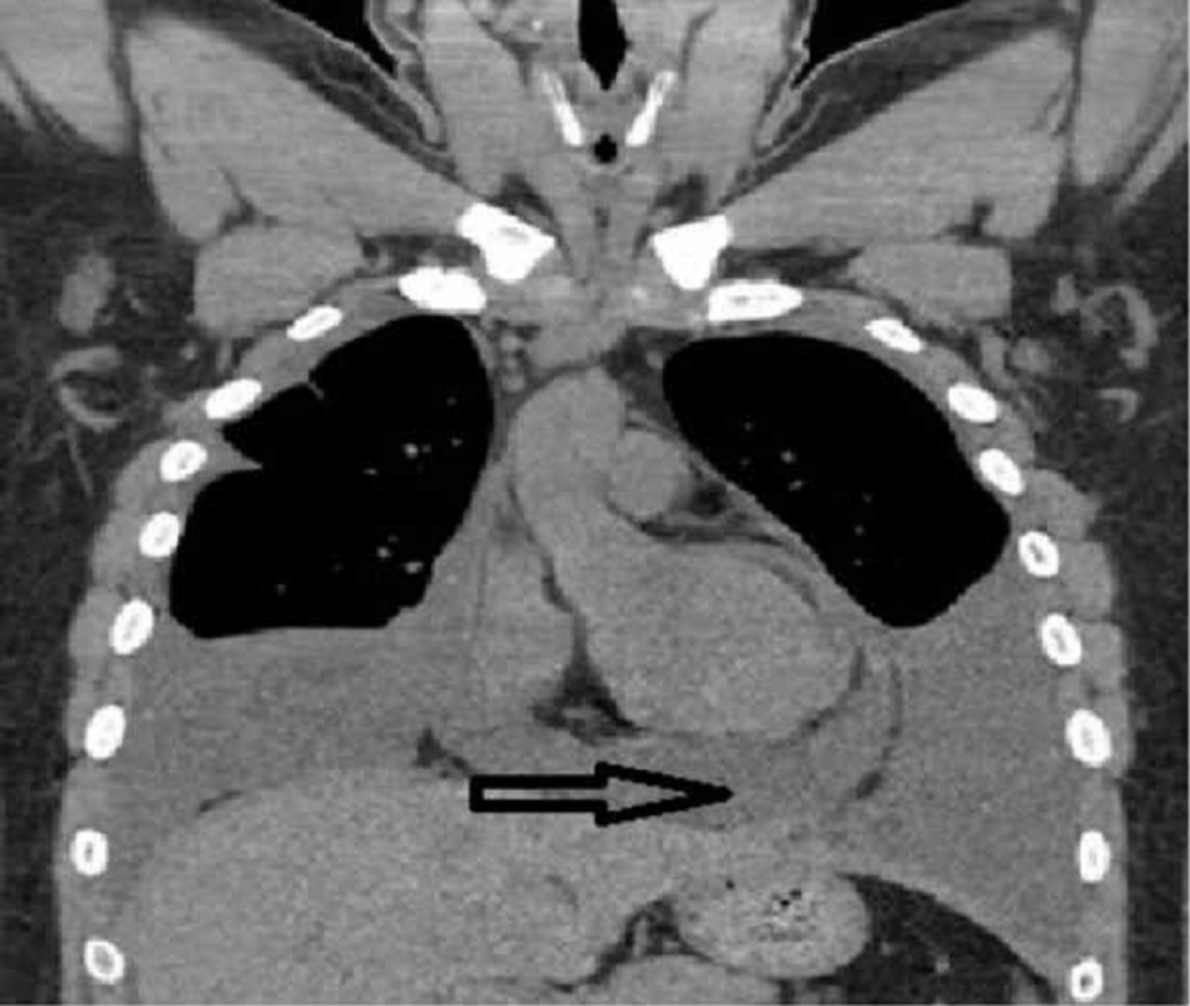
Computed tomography of chest (coronal view) showing pericardial effusion and bilateral pleural effusion Arrows pointing pleural and pericardial effusion

The patient is a 22-year-old male, chronic smoker, with no significant past medical history, with recent pneumonia associated with pleural and pericardial effusion, treated four weeks ago. He presented to the emergency department with recurring exertional dyspnea, chest pain, dry cough, and chills. The patient has a family history positive for mother having lupus and hypothyroidism. The initial chest x-ray showed cardiomegaly and bilateral recurrent pleural effusion. His d-dimer was elevated at 2.81 (0.19-0.50 mg/l) with negative bilateral lower extremity venous doppler. He had a computed tomography angiography (CTA) of the chest, which was negative for pulmonary emboli but showed large pericardial and bilateral pleural effusions (Figures 1-3). Electrocardiography (EKG) showed sinus rhythm and troponins were negative. Labs were not suggestive of any infection (Table 1). Urinalysis negative for proteinuria and urine protein/creatinine ratio not suggestive of lupus nephritis. Immunological work-up showed homogenous pattern antinuclear antibodies (ANA) and positive anti-double-stranded DNA, with normal complement levels and liver function tests. Rheumatoid factor, anti-RNP (ribonuclear protein antibody), anti-Jo, and anti-Sm antibodies were negative ruling out other etiologies. Thyroid-stimulating hormone (TSH) was elevated at 134 (0.358-3.740 uIU/ml), low T4 0.12 (0.76-1.46 ng/dl) with low T3 <0.5 (2.18-3.98 pg/ml). Thyroid peroxidase (TPO) and thyroglobulin antibodies were positive suggestive of autoimmune Hashimoto's thyroiditis. No goiter was noted on the clinical exam. The patient's presentation was consistent with an overlap of SLE with serositis and Hashimoto's thyroiditis. Cardiothoracic surgery was consulted, and the patient had bilateral chest tubes insertion with pericardial window placement. The patient was started on tapering steroid therapy along with levothyroxine supplementation. Cytology from the pericardial fluid showed reactive cells with scant inflammation and was negative for any malignancy. He had good clinical improvement with the eventual removal of the chest tubes. The patient was advised to follow up with rheumatology and endocrinology after discharge. Post-discharge follow up showed continued clinical improvement.

**Table 1:** Laboratory findings ESR - erythrocyte sedimentation rate; CRP - C-reactive protein; ANA - anti-nuclear antibody, Anti-DsDNA - anti-double-stranded DNA; C3, C4 - complement levels 3, 4; LFT - liver function tests; RF/Anti-CCP - rheumatoid factor/cyclic citrullinated peptide; Anti-RNP - anti ribonucleo protein; TSH - thyroid stimulating hormone; T3, T4 - thyroid hormone 3, 4; TPO - thyroid peroxidase

Laboratory findings	
ESR	52 (0-15mm/hr)
CRP	76 (<3.0 mg/l)
ANA	Positive homogenous pattern. 1:320 (normal <1:80)
Anti-DsDNA	65 (0-9)
C3 levels	141 (82-167 mg/dl)
C4 levels	18 (14-44 mg/dl)
LFT’s	Normal limits
RF/Anti-CCP	13 (<15 IU/ml)/ 11 (0-19)
Anti RNP	<0.2
Anti-Jo	<0.2
Anti-Sm	<0.2
TSH	134 (0.358-3.740 uUI/ml
T3	<0.5 (2.18 -3.98 Pg/ml)
T4	0.12 (0.76-1.46 ng/dl)
TPO	245 (0-34 IU/ml)
Thyroglobulin	352 (0-0.9 IU/ml)
Urine protein/creatinine ratio	5.6 (0-200)
Urine microalbumin/creatinine ratio	5.6 (0-30)

## Discussion

SLE is one of the autoimmune disorders with variable presentation and multisystem involvement. Sometimes it can present in conjugation with other autoimmune disorders, which are underdiagnosed due to overlap with clinical and radiology findings [2, 3]. Autoimmune thyroiditis, especially Hashimoto's, is one such manifestation. Patient with SLE are more prone to thyroid problems compared to the controls and is usually more so in those having overlap syndrome [4, 5]. They can have positive thyroid antibodies with or without overt thyroid manifestations [6]. it predominantly manifests in patients with SLE and Hashimoto's during the hypothyroid state rather than hyperthyroid, suggesting that the initial hyperthyroid state could be obscured [7]. Pericardial effusion is one of the common cardiac manifestations of the hypothyroid state of Hashimoto's [8-10]. Our case highlights the patient whose hypothyroidism was subclinical and undiagnosed until the severe manifestations were evident [11]. LE (lupus erythematosus) cell detection assay used to be done in the past, which is sometimes seen in effusions from SLE, which can help differentiate SLE from other causes of pericardial effusion [12]. LE cell detection may be especially encountered with an uncommon presentation [13].

## Conclusions

Patients with serositis need to be evaluated for other coexistent autoimmune disorders. This case highlights autoimmune hypothyroidism that can manifest as pericardial effusion, which needs to be considered when treating patients with SLE and pericarditis. LE cell detection in the fluid analysis may help differentiate SLE from other causes of serositis in some of the cases. Patients with untreated hypothyroidism are slow to respond to standard SLE treatment.
